# Efficient algorithms for fast integration on large data sets from multiple sources

**DOI:** 10.1186/1472-6947-12-59

**Published:** 2012-06-28

**Authors:** Tian Mi, Sanguthevar Rajasekaran, Robert Aseltine

**Affiliations:** 1 Department of Computer Science and Engineering, University of Connecticut Storrs, Connecticut, USA; 2 Institute for Public Health Research, University of Connecticut, East Hartford, Connecticut, USA

## Abstract

**Background:**

Recent large scale deployments of health information technology have created opportunities for the integration of patient medical records with disparate public health, human service, and educational databases to provide comprehensive information related to health and development. Data integration techniques, which identify records belonging to the same individual that reside in multiple data sets, are essential to these efforts. Several algorithms have been proposed in the literatures that are adept in integrating records from two different datasets. Our algorithms are aimed at integrating multiple (in particular more than two) datasets efficiently.

**Methods:**

Hierarchical clustering based solutions are used to integrate multiple (in particular more than two) datasets. Edit distance is used as the basic distance calculation, while distance calculation of common input errors is also studied. Several techniques have been applied to improve the algorithms in terms of both time and space: 1) Partial Construction of the Dendrogram (PCD) that ignores the level above the threshold; 2) Ignoring the Dendrogram Structure (IDS); 3) Faster Computation of the Edit Distance (FCED) that predicts the distance with the threshold by upper bounds on edit distance; and 4) A pre-processing blocking phase that limits dynamic computation within each block.

**Results:**

We have experimentally validated our algorithms on large simulated as well as real data. Accuracy and completeness are defined stringently to show the performance of our algorithms. In addition, we employ a four-category analysis. Comparison with FEBRL shows the robustness of our approach.

**Conclusions:**

In the experiments we conducted, the accuracy we observed exceeded 90% for the simulated data in most cases. 97.7% and 98.1% accuracy were achieved for the constant and proportional threshold, respectively, in a real dataset of 1,083,878 records.

## Background

Increased use of electronic medical record systems and the development of health information exchanges have enormous potential to expand our understanding of the health of diverse patient populations. These efforts would be greatly augmented by the capacity to integrate patient medical records with disparate public health, human service, and educational databases, which usually do not have a universal identifier. For instance, health agencies may be interested in the integrated patients’ records across multiple hospitals to track complete patients’ health histories, to reveal mechanisms behind certain diseases, or to find reasons for local diseases. Data integration techniques for identifying records belonging to the same individual that reside in multiple datasets, in the absence of any universal identifier,can improve health observation [1], improve injury surveillance systems, and health policy decisions [2] etc. It is also applied in health care insurance claims [3] and linkage of patients and health test results [4]. Data integration is also used in other areas, such as similarity detection of digital files and document fingerprinting [5,6]. Typically, integration of datasets is done without the existence of a global identifier [State of Connecticut. Connecticut Health Information Plan: A roadmap for improving access to health data. 2009 May. (unpublished report)]. Since errors might have been introduced while entering information, prediction of a record’s owner can never be *100%* correct.

When there are only two data sets *A* and *B*, the data integration problem is to decide if the records *a* and *b* belong to the same person (for every $\left(a,b\right)\in A\times B)$. Traditional approaches compare each record pair iteratively and generate a comparison vector. As a result, each record pair is classified as a true match, possible match, or true non-match. Usually a learning algorithm is employed for this classification [7]. The data integration problem that we are facing is much more challenging: 1) the records are from two or more sources; 2) the total number of records is quite large (a million or more) with very limited number of attributes to compare, and 3) the same dataset may have multiple records for individuals.

To apply the traditional method on multiple data sets may have some difficulties when dealing with the above challenges: 1) The cross-product of the datasets could be prohibitively large. For instance, consider ten datasets with two records in each. The number of 10-tuples is *2*^*10*^ = *1024*. If we have *q* datasets consisting of *n*_1_, *n*_2_, …, *n*_*q*_ records, respectively, then the total number of q-tuples to be enumerated is $n_{1}\times n_{2}\times \ldots ,\times n_{q}$ . Therefore, it is impractical to process large datasets. 2) One may want to ignore tuples and still process based on pairs. However this may lead to difficulties such as the pair *(A, B)* are decided to be matched while the pairs *(A, C)* and *(B, C)* being decided to be non-matched. 3) If tuples being used, redundant computation happens frequently when comparing records, for instance, records *A* and *B* may appear many times in different tuples and therefore in different tuples repeated computation on *A* and *B* may be done. And memory could be a bottleneck if we generate all possible tuples. Also, if pairs are used, the classification may suffer from poor accuracy. For example, a simplistic approach to matching three or more data sets involves matching two data sets iteratively, with the result of matching the first two data sets used as input for matching with the third data set, and so on. However, with this approach the final results are highly sensitive to the ordering of the datasets. Because of these disadvantages, the traditional probabilistic model seems to be inadequate for integrating multiple data sets.

Therefore, in this paper, we propose a new model to integrate multiple datasets. We employ hierarchical clustering [8] and avoid the computation of cross-products. Currently we use two methods to deal with common errors in the input data, typing distance and sound distance. But other comparison methods can easily be added into our model. Besides, our algorithms also take care of the following types of errors: reversal of the first and last names, nickname usage, and attribute truncation. Our algorithms are both time and space efficient. The excellent results from our experiments show that our clustering model is robust and promising when dealing with large data. With our model, new records can be easily inserted into old results without any re-computations on the old data once the old data dendrogram from Hierarchical Clustering is stored.

## Methods

### Previous approaches

Traditional approaches mainly involve two steps: comparison of record pairs to generate comparison vectors, and classification of record pairs into three sets of true match, possible match, and true non-match based on the comparison vectors [9,10]. A probabilistic model was introduced by Fellegi and Sunter in 1969 [11], in which comparison only considers match/non-match values. A lot of studies have been done following the probabilistic model [12-14]. Expectation maximization (EM) algorithm has been used to get better decision rules when maximum likelihood is reached in [15]. While a relational probability model was used for citation matching [16], conditional models have been proposed to capture the dependency features under certain background, for instance, using conditional random fields in context [17,18]. Based on such conditional models, deduplication has been studied in relational databases of different data types, e.g. information of an entire record is kept in different data tables [19,20]. This probabilistic model can also support categorical comparison values [14]. Several continuous-value comparisons also appear to deal with typographic errors [21], such as Hamming Distance, Edit Distance [22], Soundex Code and Metaphone (http://www.sound-ex.com/alternative_zu_soundex), Jaro-Winkler Comparison [23], Q-grams (or N-grams) [24], Longest Common Substring [25], and so on.

Several software and packages have also been developed to solve this record linkage problem. FEBRL is one of the excellent ones, which employs many existing techniques and can handle datasets with several hundred thousand records but with no clear accuracy and time analysis [26-28], together with TAILOR [29], IntelliClean [30], Merge/Purge [31] and so on. Most of these algorithms pre-process the linkage with a blocking phase, hashing by a blocking key [29], for instance. Sorted-Neighborhood Method (SNM) [31,32] limits the comparison within a window after sorting the data, which is used as blocking by FEBRL [26-28], TAILOR [29], and IntelliClean [30]. Canopy Clustering [33] is also used as a blocking method in FEBRL [26-28]. However, not much work has been done to integrate multiple datasets. We propose a novel technique based on hierarchical clustering. Clustering partitions a set of observations into subsets (called clusters) such that observations in the same cluster are similar. Clustering has been applied in many fields such as data mining [34,35], machine learning [36], psychology [37], and even genetics [38-41]. Hierarchical clustering creates clusters in a tree structure (called a dendrogram) (see e.g., [42-45]). Various parallel algorithms for it have also appeared (see e.g., [46-48]). Given a set of n points to be clustered, the agglomerative hierarchical clustering starts with n clusters where each cluster has a single input point with a distance of *0*. From there on, the algorithm proceeds in stages. In each stage the two closest clusters are merged into one and the distance of this new cluster is the distance between the merged clusters. The stages of merging happen until we are left with one cluster (containing all the input points). We can cut the tree at any desired threshold level. Note that each cluster is associated with a distance. All the root clusters at the threshold level will be output by the algorithm. Any hierarchical clustering algorithm can be classified based on the distance measure used. In this paper we use the single linkage clustering. It has been shown that results are similar using different linkage in Hierarchical Clustering [49], and single linkages has the advantage in time complexity [47].

### Basic methodology

Our approach for data integration is to treat each record in each data set as a point in a multi-dimensional space. The dimension of this space is decided by the number of attributes in the records. We then define an appropriate distance measure for these points. Followed by this, we cluster these points and the clusters lead us to the identification of common records.

### Record distance calculation

In this paper, we consider attributes such as the first name, last name, gender, zip code, etc. However, the techniques described are generic and should be applicable for any set of attributes. We define the distance between two records in a number of ways by appropriately combining attribute distances. We use *RD(R*_*1*_*,R*_*2*_*)* to denote the distance between two records *R*_*1*_ and *R*_*2*_. Given *R*_*1*_ and *R*_*2*_ from any data sets, the common attributes of the two records are used to calculate *RD(R*_*1*_*,R*_*2*_*)*.

*Definition 1: Assume that each of the two records R*_*1*_*and R*_*2*_*from any data set have n common attributes and let d*_*1*_*, d*_*2*_*,…, and d*_*n*_*be the attribute distances between these records. Then, RD(R*_*1*_*,R*_*2*_*) is defined as d*_*1*_ *+ d*_*2*_ *+ … + d*_*n*_*. Here d*_*i*_*is the distance between the i*^*th*^*attribute of R*_*1*_*and the same attribute of R*_*2*_*.* In this paper, the combination operator we consider is addition for simplicity, but other methods, like giving different weights to different attributes could be easily plugged in.

We consider two kinds of input errors, based on typing errors and sound similarity. We use the edit distance as the basic distance measure for the typing errors, while Metaphone is used to deal with errors based on sound similarity. The Edit Distance (also known as the *Levenshtein Distance*) between two strings is the minimum number of operations, such as deletion, insertion, and exchange that are required to transform one string to the other [22]. One lower bound on the edit distance between two strings is the difference in the lengths of the two strings. One could use dynamic programming to compute the edit distance between two strings of length *n* and *m*, respectively, in *O(mn)* time (see e.g., [50]). With the Four-Russians speedup, which partitions the dynamic programming table into blocks and looks up the distance of each block from a pre-computed block offset function, the edit distance can be computed in *O(l*^*2*^*/log(l))* time when an appropriate block size is chosen (where *l* is the length of the two strings) [51]. In this paper, we assume a penalty of *1* for each of the operations insertion, deletion, and exchange. Given two attributes *A*_*1*_ and *A*_*2*_, the edit distance of *(A*_*1*_*,A*_*2*_*)* is the attribute distance based on edit distance. We incorporate into edit distance several methods to deal with common typing errors, such as 1) Reversal of the first name and the last name (*reversal distance*): In this case we use the smaller of the distance between the names and the distance between one name and the reversal of the other; 2) The use of nicknames (*nickname distance*): In this case we look up a nickname table (http://www.cc.kyoto-su.ac.jp/~trobb/nicklist.html) and use the smallest edit distance; and 3) Truncation of attributes (*truncation distance*): only consider the first few letters. Given two names, the *name distance* between them is defined as the smallest distance obtained from the names’ edit distance, reversal distance, nickname distance, and truncation distance. Errors based on pronunciation or sound similarity is another main issue when data is input. Metaphone is a phonetic algorithm to encode strings based on phonetic similarity, which works more effectively than the Soundex Coding. *Phonetic distance* between two attributes is defined as: 1) zero, if the Metaphone codes of them are the same; 2) edit distance of the Metaphone codes, otherwise.

### The basic algorithm

We employ hierarchical clustering to deal with the problem of integrating multiple (more than two) datasets. Our basic algorithm (Algorithm 1) is to take every record in each data set as a point (in an appropriate space). Single linkage hierarchical clustering is applied on these points. The clustering yields a dendrogram. A relevant threshold is employed to cut this dendrogram to obtain clusters of interest. In Step 2, a threshold is needed to collect the required clusters. We provide two types of thresholds - constant and proportional thresholds. The constant threshold allows a certain number of errors occurring in record comparisons, while the proportional threshold requires the number of errors to be limited to a percentage of the total record length.

Algorithm 1: Basic integration algorithm (BIA)

Step 1. Construct the dendrogram using hierarchical clustering.

Step 2. Cut the dendrogram at the level of a threshold. Collect the root clusters at the threshold level and output.

Ideally, each cluster will only have points corresponding to the same person, i.e., all the versions of the same record will be in one cluster and this cluster will not have any other records. However in practice this may not always be the case. Thus we characterize the performance of this technique with an accuracy parameter (See RESULTS Section).

If *l* is the maximum length of any record, the distance between two records can be computed in *O(l*^*2*^*)* time. Therefore, when the total number of records is *n*, Steps 1 and 2 need *O(n*^*2*^*l*^*2*^*)* time and *O(n*^*2*^*)* space.

### Improved algorithms

Algorithm 1 takes too much time and space even on reasonably small data sizes. Note that the time and space requirements of Algorithm 1 are quadratic in the total number of records. Data sets of practical interest have millions of records. Table 1 displays the performance of Algorithm 1 on small datasets. Extrapolating from the numbers shown in this table, we can expect that Algorithm 1 will take around 20 hours when the number of records is *100,000*. To overcome the shortcomings of Algorithm 1, we have employed a series of techniques to improve its performance. In this section we describe these techniques.

**Table 1:** Experimental results on simulated data sets (constant threshold)

	**Algorithm**	**Com**	**Acc**	**Time(ms)**	**Com**	**Acc**	**Time(ms)**	**Com**	**Acc**	**Time(ms)**
	BIA	99.4%	98.8%	14702	98.5%	97.0%	342411	-	-	-
	PCD	99.4%	98.8%	12422	98.5%	97.0%	334583	-	-	-
*RDED*	IDS	99.4%	98.8%	11562	98.5%	97.0%	291162	99.7%	99.3%	1200810
*t = 30*	IDS(FCED)	99.4%	98.8%	6031	98.5%	97.0%	164307	99.7%	99.3%	693665
	TPA	92.2%	78.3%	406	90.7%	69.9%	7703	97.7%	91.8%	39843
	TPA(FCED)	92.2%	78.3%	265	90.7%	69.9%	5266	97.7%	91.8%	21640
	BIA	99.4%	98.8%	14453	98.3%	97.0%	354332	-	-	-
	PCD	99.4%	98.8%	13812	98.3%	97.0%	360613	-	-	-
*NDED*	IDS	99.4%	98.8%	13859	98.3%	97.0%	317052	99.6%	99.4%	1351910
*t = 30*	IDS(FCED)	99.4%	98.8%	9016	98.3%	97.0%	204071	99.6%	99.4%	861785
	TPA	92.2%	78.3%	469	90.7%	70.0%	9484	97.7%	91.8%	42436
	TPA (FCED)	92.2%	78.3%	359	90.7%	70.0%	6140	97.7%	91.8%	27155
	BIA	98.8%	98.8%	11000	96.9%	96.4%	301960	-	-	-
	PCD	98.8%	98.8%	11234	96.9%	96.4%	299756	-	-	-
*PDED*	IDS	98.8%	98.8%	10547	96.9%	96.4%	254805	98.8%	98.9%	1046654
*t = 30*	IDS(FCED)	98.8%	98.8%	5390	96.9%	96.4%	145604	98.8%	98.9%	587013
	TPA	92.4%	78.6%	391	90.7%	69.9%	7516	97.7%	91.8%	33499
	TPA(FCED)	92.4%	78.6%	235	90.7%	69.9%	4313	97.7%	91.8%	20312
Size		1,000			5,000			10,000		

### Partial construction of the dendrogram

Instead of building the entire dendrogram and cutting at the threshold level, the idea here is to construct only portions of the tree that are below the threshold level. The resultant algorithm is shown as Algorithm 2.

Algorithm 2: PCD

Step 1. Collect all the records in all the data sets. Each such record is a cluster by itself at level 0 initially.

Step 2. Calculate the record distances between each pair of clusters. Keep a nearest neighbor for each cluster.

Step 3. Find the pair of clusters with the smallest distance.

Step 4. If this distance is smaller than or equal to the threshold, then merge the two clusters into a new cluster with the distance as the new cluster’s level. Otherwise, go to Step 7.

Step 5. Update the pairwise distances and the new nearest neighbors’ information.

Step 6. Repeat Steps 3, 4, and 5 until we end up with a single cluster.

Step 7. Collect and output the root clusters.

Although Algorithm 2 also takes *O(n*^*2*^*l*^*2*^*)* time, its expected run time is better than that of Algorithm 1. This is especially true if the error rate is low. If the errors in the records occur with a low probability, then the threshold value will be low and we only have to construct a small portion of the dendrogram.

### Ignoring the dendrogram structure

Since only certain clusters are collected as the output, the structure of the dendrogram is not necessary. Algorithm 3 shows the details of this technique.

Algorithm 3: IDS

Step 1. Collect all the records in all the data sets. Let this collection be *D*.

Step 2. *While D* is not empty *do*

Pick one of the records in *D* arbitrarily. Let *R* be this record. We will form a cluster *C*_*R*_ corresponding to *R* as follows. To begin with *C*_*R*_ has only *R* in it. We identify all the records in *D* that are at a distance of ≤ *threshold* from *R*. Let the collection of these records be *C’*. Add all the records of *C’* to *C*_*R*_. Followed by this identify all the records of *D* that are at a distance of  ≤ *threshold* from any of the records of *C’*. Let the collection of these records be *C”*. Add all the records of *C’*’ to *C*_*R*_. Continue this process of identifying neighbors and adding them to *C*_*R*_ until no new neighbors can be found. At this point output *C*_*R*_. This is one of the clusters of interest. Set *D* := *D - C*_*R*_.

Since Algorithm 3 does not generate a distance matrix, its memory usage is only *O(n)* and the worst case run time is still *O(n*^*2*^*l*^*2*^*)*.

### Faster computation of the edit distance

The general algorithm for edit distance computation takes quadratic time (see e.g., [22,50]). Since we have to calculate edit distances for a total of *n*^*2*^ times, a speedup in edit distance calculation will improve the run time on the entire data integration task. The four-Russians speedup algorithm on edit distance runs in *O(l*^*2*^*/log(l))* time, when a block size is chosen to be *(log*_*3σ*_*(l))/2* (where *σ* is the alphabet size and *l* is the length of two strings). However, unfortunately, it is inapplicable due to the short length of the strings involved since in this problem, *σ* = *26* and usually *l* ≤ *50*.

Observation 1: The edit distance between two strings is always larger than or equal to the difference in the lengths of the two strings.

It is easy to see that even if one string *S*_*1*_ is a substring of the other *S*_*2*_, it still needs *|S*_*2*_*|-|S*_*1*_*|* number of inserts and/or deletes to transfer one to the other. This lower bound can help to see whether the edit distance between two strings is larger than the threshold even before the calculation of the edit distance. In this case we can omit the calculation of the edit distance.

In summary, let *t* be a given a threshold. If the distance between two strings *S*_*1*_ and *S*_*2*_ is less than or equal to *t*, then we can compute this distance in $O\left(tl\right)\text{time},\text{where}l=min\{\left|S_{1}\right|,\left|S_{2}\right|\}$.

Dynamic programming is typically used to compute the distance between two strings. This algorithm employs a table of size $l\times l(\text{where}l=max\left\{\left|S_{1}\right|,|S_{2}|\right\})$to compute partial distances (see e.g., [34]). If the edit distance between two strings is smaller than *t*, then the trace-back path of the dynamic programming table should be within a strip of size *(2 t + 1)* centered on the diagonal. The diagonal is where we align the *i*^*th*^ character of one string to the same *i*^*th*^ character of the other string. Departure from the diagonal means insertion or deletion, so when at most *t* inserts or deletions are allowed, the trace-back path is of departure at most *t* from the diagonal in a row. If the edit distance is smaller than *t*, what we calculate is the accurate edit distance. Otherwise, what we calculate is not accurate, but since we know that the edit distance is more than *t*, it does not matter that the edit distance computed is not accurate. We refer to this technique of computing edit distances as FCED (fast computation of edit distances). This idea is also described in Gusfield's book ([52] 261–262). In particular, in any row, column, or diagonal of the dynamic programming table (for edit distance computation), two adjacent cells can have values that differ by at most one ([52] 305–306). Also, in any diagonal of the dynamic programming matrix *D* (for edit distance computation), either $D\left[i,j\right]=D\left[i-1,j-1\right],\text{ or}D[i,j]=D\left[i-1,j-1\right]+1$. (Matrix *D* is defined as follows. If we are interested in computing the distance between two strings $X=x_{1}x_{2}\ldots x_{n},\text{ and}Y=y_{1}y_{2}\ldots y_{m}$ using dynamic programming, then a matrix of size *n*×*m* will be employed. In particular, *D[i,j]* will be the distance between $x_{1}x_{2}\ldots x_{i}\text{and}y_{1}y_{2}\ldots y_{j}$ (for all values of i and j)). We can see that $D\left[i,j\right]\neq D[i-1,j-1]-1$ , then $D\left[i,j\right]$ is calculated from $D\left[i-1,j\right]\text{or }D[i,j-1]$. Without loss of generality, assume that $D\left[i,j\right]=D\left[i-1,j\right]+1.$ Then $D\left[i-1\text{,}\;j-1\right]=D\left[i,j\right]-2$, which is a contradiction.

This fact implies that the values along the diagonal of the dynamic programming table are non-decreasing.

Using Observation 1, we first check if the edit distance is larger than the threshold. If not, we employ the *O(tl)* time algorithm as described above. We keep checking the diagonal to see whether the allowed threshold *t* is broken through. If the edit distance is seen to be larger than the threshold, the algorithm terminates immediately. Otherwise, the accurate edit distance is calculated and returned. In this algorithm, the threshold is treated as the current distance allowed. While dynamically calculating the edit distance, the diagonal value is compared with the threshold. If the value is already larger than the threshold, no further calculation is necessary and an infinite distance is given for such comparisons. If there is another pair of attributes to compare, a new distance allowed is computed as the threshold minus the distance already used. This method is implemented in Algorithm 3, and Algorithm 4 (in the next section).

### A two-phase algorithm

Now we describe a two-phase algorithm. In the first phase (called *a blocking phase*), records sharing something in common are indexed into the same block. Records in each block will be integrated later. Blocking phase uses the *last name* attribute, which is considered more accurate than the first name.

In each record, the last name is parsed into *l-mers* (usually *3-mer* or *4-mer*), and this record is added into blocks which are indexed by these *l-mers*. For instance, in the case of *l* = *3*, ``Rueckl" is indexed into *4* blocks: ``rue", ``uec", ``eck", and ``ckl". The total number of possible blocks is *26*^*l*^. Since one record may belong to multiple blocks, after integration on each block, a post-processing is done to merge clusters with duplicate records. Details are shown as Algorithm 4.

Algorithm 4: TPA

Step 1. Collect all the records in all the datasets. Put them into blocks based on *l-mers* of the last names.

Step 2. Integrate records in each block using the algorithm IDS. Step 3. Merge the clusters with the same records.

Assuming that there are *b* blocks, integrating each block takes *O((n/b)*^*2*^ *l*^*2*^*)* time on an average. Over all the *b* blocks the expected run time is *O(n*^*2*^*l*^*2*^*/b)*. Note that *b* is typically a large number. For *3-mer* blocking, *b = 26*^*3*^ *= 17576* and for *4-mer* blocking *b = 26*^*4*^ *= 456976*.

In summary, we have proposed four algorithms for multiple-source integration, together with six distance calculations: edit distance to handle common typos, reversal distance to handle the last and first names reversal errors, nickname distance to handle the distances with nicknames, truncation distance to handle the errors with abbreviations, phonetic distance to handle the similarity of sound, and name distance to capture all features of edit, reversal, nickname, and truncation distances on names. Without loss of generality, we validated our algorithms on simulated data by 1) reversal distance for the first or last names and edit distance for the other attributes (*RDED*); 2) name distance in the first and last names and edit distance in the other attributes (*NDED*) where the truncation length is *5* for both the first and the last names; and 3) phonetic distance in the first and last names and edit distance in the other attributes (*PDED*). For real data, because of the limited common attributes (only first name, last name, and date of birth), *ED*_*name*_ calculates the edit distances based on the first and the last names, *ED*_*all*_ considers all the three attributes, *PD*_*name*_ calculates the phonetic distances based on the first and the last names, and *PDED* calculates the phonetic distances on the first and the last names and edit distance on date of birth.

## Results

We have implemented our technique in Java and tested it on simulated data sets, as well as some real datasets. Additional file 1 details the generation of the simulated data sets. Also, the real data sets come from the Connecticut Health Information Network (CHIN), which have a total of *1,083,878* records (Please see Additional file 2 for details).

### Results on simulated data

Since our algorithms collect all the records from all the input datasets, the number of input datasets is not important. The performance of our algorithms depends only on the total number of records (from all the datasets). Therefore, we generate a single input dataset for each test. Three datasets of size *1,0005,000*, and *10,000 respectively,* are generated following [53] (Please see Additional file 1 for details). The computer we have used has a *CPU of Intel(R) 2.83 GHz Core(TM)2 Quad Q9550*, with a memory of *4 GB*.

The detailed results on simulated data are shown for both constant thresholds and proportional thresholds, . In Table 1 and Table 2, three comparison methods, *RDED*, *NDED*, and *PDED,* have been used to calculate the distances among records, and all the algorithms are tested for each distance calculation. The first (Com., Acc., Time) is for data size *1,000*, the next is for *5,000*, and the last for *10,000*. We calculate the accuracy as follows. Let *N* be the number of output clusters and let *C* be the number of *correct* clusters. A cluster is correct if it has all the records pertaining to only one individual and no other records. The *accuracy* (Acc.) is then computed as *C/N* expressed as a percentage. Another metric to evaluate the performance is *completeness* (Com.), which is defined as follows. Let N* be the total number of entities or persons in all the input data sets. *Completeness* is the value of C/N*, indicating how many entities’ records are picked up correctly. In Table 1 and Table 2, thresholds were picked up by training the data set of size *1,000*, and applied to the other two data sets. The *accuracy* and *completeness* of those two data sets suggests that picking up thresholds in this training and learning method is pretty safe. From Table 1 and Table 2 (tests marked by”-” were terminated after waiting for 30 minutes), in general, most *accuracies* and *completeness* exceed 90% which indicates our approach’s capability in records matching. Especially algorithm TPA with FCED technique has a dramatic improvement in the run time, for instance, using *RDED* in *10,000* data size, IDS took *1201 s*, and IDS(FCED) took *694 s*, while TPA only took *40 s* and TPA(FCED) took *22 s*, which is roughly *30* times faster, though with some drop in the accuracy (e.g. *99.7%* of IDS(FCED) to *97.7%* TPA(FCED) in *completeness* and *99.3%* to *91.8%* in *accuracy*) due to the fact that in the blocking phase not all the records which pertain to the same person can be indexed into the same block. This small drop in the accuracy may be worthwhile, especially when dealing with large data and if time is a critical factor. As a result, we have employed TPA and TPA (FCED) on real data sets.

**Table 2:** Experimental results on simulated data sets (proportional threshold)

	**Algorithm**	**Com.**	**Acc.**	**Time(ms)**	**Com.**	**Acc.**	**Time(ms)**	**Com.**	**Acc.**	**Time(ms)**
	BIA	98.4%	96.9%	14593	97.7%	95.4%	345880	-	-	-
	PCD	98.4%	96.9%	13515	97.7%	95.4%	345645	-	-	-
*RDED*	IDS	98.4%	96.9%	11422	97.7%	95.4%	298069	99.5%	99.0%	1225476
*t = 0.35*	IDS(FCED)	98.4%	96.9%	7125	97.7%	95.4%	173932	99.5%	99.0%	673650
	TPA	91.8%	77.7%	515	90.4%	69.5%	9203	97.6%	91.7%	38499
	TPA(FCED)	91.8%	77.7%	281	90.4%	69.5%	4500	97.6%	91.7%	23374
	BIA	98.4%	96.9%	14547	97.8%	95.6%	384222	-	-	-
	PCD	98.4%	96.9%	14156	97.8%	95.6%	381191	-	-	-
*NDED*	IDS	98.4%	96.9%	13671	44.6%	99.6%	343927	99.6%	99.1%	1416142
*t = 0.35*	IDS(FCED)	98.4%	96.9%	9078	44.6%	99.6%	222305	99.6%	99.1%	884472
	TPA	91.8%	77.7%	485	42.5%	90.5%	10140	97.6%	91.7%	45436
	TPA(FCED)	91.8%	77.7%	344	42.5%	90.5%	6484	97.6%	91.7%	29030
	BIA	98.6%	97.2%	11890	97.8%	95.5%	314115	-	-	-
	PCD	98.6%	97.2%	12046	97.8%	95.5%	313006	-	-	-
*PDED*	IDS	98.6%	97.2%	11031	97.8%	96.0%	272085	99.6%	99.1%	1083059
*t = 0.35*	IDS(FCED)	98.6%	97.2%	5937	97.8%	96.0%	165495	99.6%	9.1%	610262
	TPA	91.8%	77.7%	250	90.1%	70.0%	7843	97.6%	91.7%	32827
	TPA(FCED)	91.8%	77.7%	171	90.1%	70.0%	4297	97.6%	91.7%	21046
Size		1,000			5,000			10,000		

To validate the thresholds chosen in training, a 5-fold cross validation was used: partition the *10,000* records into *5* equal sets and randomly pick four sets as the training data to get the thresholds and the remaining as the testing data, and repeat *5* times. Table 3 shows the result of the 5-fold cross validation. Accuracies obtained (around ~98%) suggest that the thresholds identified in training are able to capture the general features of the data and therefore separate records of the same person from the others pretty well.

**Table 3:** Accuracies in 5-fold cross validation on picking up the threshold

	**model1**	**model2**	**model3**	**model4**	**model5M**
constant = 30	99.3%	99.3%	99.7%	99.6%	97.3%
proportion = 0.35	99.2%	99.1%	99.4%	99.0%	97.0%

We also tested on a large dataset with *1,000,000* records. Results in Table 4 show the efficiency and robustness of the proposed algorithms.

**Table 4:** Experimental results on real data sets (N = 1,083,878)

		**Time - TPA**	**Time – TPA(FCED)**	**Clusters**	**Acc. Clusters**	**Individuals**	**Acc.**	**Com.**
*ED*_*name*_	constant t = 1	1:52:41	0:27:29	94,381	87,756	108,800	93.0%	80.7%
*ED*_*all*_	t = 1	3:11:17	0:29:33	101,864	99,562	108,800	97.8%	91.6%
*PD*_*name*_	-	1:06:04	1:04:13	90,950	83,270	108,800	91.6%	76.5%
*PDED*	t = 1	2:04:09	1:06:04	101,344	99,711	108,800	98.4%	91.6%
*ED*_*name*_	proportional t = 0.1	1:55:24	0:30:56	94,521	87,966	108,800	93.1%	80.9%
*ED*_*all*_	t = 0.1	3:14:37	0:44:05	101,254	99,346	108,800	98.1%	91.3%
*PD*_*name*_	-	1:04:32	1:05:41	90,950	83,270	108,800	91.6%	76.5%
*PDED*	t = 0.1	2:06:16	1:09:02	100,896	98,949	108,800	98.1%	90.9%

### Results on real data

We have a total of *1,083,878* records and multiple records from one person in each of the four databases are common (Please see Additional file 2 for detail). A linkage gold- standard has shown that the attribute combination of Social Security Number, phonetically compressed first name, birth month, and gender is the best one to find record linkage [54]. However, the common attributes across all the 4 data sets were very limited: first name, last name, and date of birth, which increased the challenge to do the integration. *ED*_*name*_ calculates the edit distances based on the first and the last names, *ED*_*all*_ considers all the three attributes, *PD*_*name*_ calculates the phonetic distances based on the first and the last names, and *PDED* calculates the phonetic distances on the first and the last names and edit distance on date of birth. The thresholds are obtained from training *4,000* records, *1,000* from each database. The detailed results are shown in Table 4. Accuracy is estimated by the combination of Social Security Number and the internally assigned DDS identification number. Out of the total *1,083,878* records, *896,174* records have valid identifiers so the analysis is based on the *896,174* records. While looking into the details of the results of *ED*_*name*_, we found that most of the inaccurate clusters resulted when tautonyms exist, i.e., when there were records with exactly the same first and last names pertaining to different persons. *97.8%* accuracy was achieved immediately when edit distance was used on all the three attributes for the constant threshold (*98.1%* for the proportional threshold) within around *30* minutes (1 hour), and *98.4%* accuracy was received for *PDED* (*98.1%* for the proportional threshold) around *1* hour, where *completeness* is above *90%* when all the three attributes were used, as shown in Table 4. In particular, the algorithm *TPA (FCED)* was about four to six times as fast as the algorithm *TPA* using edit distance, and even with edit distance of one attribute *TPA (FCED*) still speeds up about two times (*PDED* in Table 4). The notion of negative data is unclear to this multiple data integration problem and hence sensitivity and specificity analysis cannot be done. However, we perform a similar analysis to look further into our results. A four-category analysis is proposed. Any cluster of records is categorized into four: 1) (Type I), if a cluster contains only one person's records and contains all of this person's records; 2) (Type II), if a cluster contains only one person's records but not all of this person's records; 3) (Type III), if a cluster contains all the records of one person but also contains some other person's records; 4) other cases can be seen as errors (Type IV). Table 5 shows the results of this analysis. Type II clusters are nothing but incomplete clusters which still play a valuable role to people. Type III clusters are similar but a little less important. Therefore, only Type IV clusters are “true incorrect”. When using all the three attributes, this “true incorrect rate” is limited within about *0.6%*.

**Table 5:** Four-category analysis on real data sets (N = 1,083,878)

		**Type I**	**Type II**	**Type III**	**Type IV**
*ED*_*name*_	constant t = 1	93.0%	2.2%	0.0%	4.8%
*ED*_*all*_	t = 1	97.7%	2.1%	0.0%	0.2%
*PD*_*name*_	-	91.6%	1.7%	0.0%	6.7%
*PDED*	t = 1	98.4%	1.3%	0.0%	0.3%
*ED*_*name*_	proportional t = 0.1	93.1%	2.2%	0.0%	4.7%
*ED*_*all*_	t = 0.1	98.1%	0.1%	0.0%	0.4%
*PD*_*name*_	-	91.6%	1.7%	0.0%	6.7%
*PDED*	t = 0.1	98.1%	1.3%	0.0%	0.6%

### Comparison with the probabilistic model using FEBRL

FEBRL [26-28] is excellent for data linkage, which exploits most of the current techniques of indexing/blocking, comparison, and classification.

Linkage of two datasets is compared in Table 6. Since any of the distance calculations of the proposed approach considers certain common errors, like insertion, deletion, and so, to be fair, we chose to use a similar distance calculation, the edit distance, in FEBRL, and we chose “FellegiSunter” from the classification methods as the probabilistic model to compare. From the real data, we randomly picked *1,000 vs. 1,0002,000 vs. 2,000*, and *3,000 vs. 3,000* data sets as three groups of linkage tests. We used *IDS (FCED)* as the non-blocking algorithm and *TPA (FCED)* as a blocking algorithm in the comparison. For each algorithm, we used *ED*_*name*_*ED*_*all*_, and *NDED* as the distance calculation. In FEBRL [26-28], only the best result was selected for a non-blocking algorithm and a blocking algorithm. It was found that the best results could be reached with *0.5* as the edit distance thresholds, respectively, and *0.3* and *0.8* as lower and upper threshold for “FellegiSunter”, and with blocking the fastest index method can be “CanopyIndex” with canopy method “Jaccard” and global tight and loose thresholds of *0.8* and *0.3* and *3* as the length of q-grams. Table 6 shows that the two approaches have similar accuracies. However, our approach takes less time for both non-blocking and blocking algorithms demonstrating the robustness of our algorithms in handling large input datasets. For FEBRL with no blocking on 3000 *vs.* 3000, we terminated the program after waiting for 15 minutes with no response, since all the other methods can finish within around 20 seconds.

**Table 6:** Performance comparison with FEBRL

	**Acc.**	**Time(ms)**	**Acc.**	**Time(ms)**	**Acc.**	**Time(ms)**	**Comments**
	100.0%	766	100.0%	3766	100.0%	8735	*DSI(FCED) + EDname*
	99.0%	2125	100.0%	11171	100.0%	15922	*DSI(FCED) + EDall*
Our	99.0%	2563	98.2%	9172	97.7%	20391	*DSI (FCED) + NDED*
Algorithms	100.0%	187	100.0%	250	100.0%	469	*TPA(FCED) + EDname*
	100.0%	234	100.0%	453	100.0%	828	*TPA(FCED) + EDall*
	99.2%	203	98.4%	516	98.0%	1047	*TPA (FCED) + NDED*
FEBRL	100.0%	40438	100.0%	173597	-	>15 min	no blocking
	100.0%	1284	100.0%	2284	100.0%	3265 s	With blocking
Size	1000 *vs.* 1000		2000 *vs.* 2000		3000 *vs.* 3000		

## Discussion

**Figure 1:**
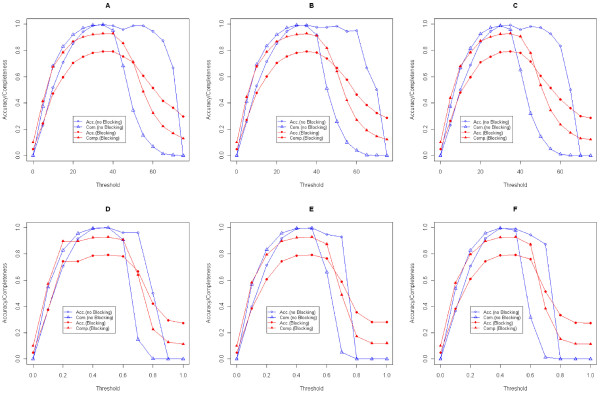
**Relationship between thresholds and accuracy/completeness. (A)***ED*_name_ with constant thresholds; **(B)***RDED* with constant thresholds; **(C)***NDED* with constant thresholds; **(D)***ED*_name_ with proportional thresholds; **(E)***RDED* with proportional thresholds; **(F)***NDED* with proportional thresholds.

To achieve a good accuracy, a good threshold value to cut the dendrogram of the hierarchical clustering is needed. We show the relationship between *Accuracy*/*Completeness* and thresholds in Figure 1. A decent threshold is needed to get a nice accuracy or completeness, which depends on many parameters such as attributes compared, attribute distances applied, combination operation used, and even the specific data sets, and should be decided empirically. In this paper we provide some guidelines for picking an appropriate threshold value.

Using a training phase is always a good method to learn the threshold. We can think of the entire process of data integration as consisting of two main processes. The first process is the training phase wherein we will be given records with identifiers to indicate which of them belong to the same person. In this phase we learn the values of all the underlying parameters (threshold, in particular). Once we learn the values of the parameters, in the second phase we can work on any (unknown) collection of datasets and integrate them. If the dataset given in the training phase is truly representative of the real world data, then the accuracy in the second phase will be high. This is typically true for any learning technique. The learning technique can only be as good as the training dataset. For example, in our experiments, for the simulated data test, the threshold was learned from datasets of size *1,000* (Table 1 and Table 2), and for real data test, the threshold was learned from *4,000* records, *1,000* from each of the four data sets. Both of these training phases resulted in good results. Another way is to use the knowledge about the input datasets to get a threshold empirically. Generally speaking, the real world data may not have too many errors and a small threshold is always suggested. In our real data experiment, one million records are more than enough to suggest that the constant threshold of *1* and the proportional threshold of *0.1* would be promising.

Also, one may want to run the application multiple times with different thresholds. With the nature of Hierarchical Clustering, it is not necessary to re-calculate everything. If the whole *dendrogram* is kept, different thresholds are just different levels to cut the tree structure and the result can be immediately output. Although a lot of studies have been made in record linkage, work has seldom been done on multiple data sets as relatively large as we discussed here. Merge/Purge is capable to handle millions of records in the parallel implementation [31]. Also, this is done by its simple clustering method of two phases, of which the first phase clusters data on an n-attribute key and the second phase applies the sorted-neighbourhood method within each cluster. Then further processes and decisions are made. Such simple clustering method supports the capability of Merge/Purge to handle large data sets fast. Though at very low possibilities coincident of errors in these n-attribute keys may risk the general accuracy, it does not harm the good tradeoff between time and accuracy in Merge/Purge since it may happen at very small probabilities. We use distance calculations to plug in the hierarchical clustering method, in the expectation that different calculation methods can be easily added into our approach and performance can be improved by new excellent calculation methods. And the efficiency is obtained by algorithm improvement within hierarchical clustering and the distance calculation.

## Conclusions

The ability to integrate diverse medical and public health datasets, particularly in this time of burgeoning availability of data from health information exchanges, offers unprecedented opportunities for health research and surveillance. A prerequisite for this, however, are techniques that allow for the simultaneous integration of multiple datasets that lack a shared numeric identifier. In this paper, we have presented an approach for the integration of records from multiple datasets. We improved our basic idea based on several different methods and implemented and experimentally validated our approach. In addition to the standard attribute distance measures, we have also introduced attribute distances based on prior knowledge of commonly occurring mistakes. Hierarchical clustering is the basis of our approach. The accuracy we have obtained is very good indicating that our approach is promising.

## Competing interests

All authors declare that they have no competing interests.

## Authors’ contributions

TM contributed to the implementation of the algorithms, testing and analysis on the synthetic and real data, manuscript preparation, algorithms development, and performance analysis. SR contributed to algorithms development, analysis of the results, performance analysis, and manuscript preparation. RA contributed to data preparation, results analysis, and performance analysis. All authors read and approved the final manuscript.

## Pre-publication history

The pre-publication history for this paper can be accessed here:

http://www.biomedcentral.com/1472-6947/12/59/prepub

## Supplementary Material

Additional file 1 **Detailed generation of the simulated data**[53]. Click here for file

Additional file 2 Introduction to the real data. Click here for file
