# Supplementary material for: Efficient algorithms for fast integration on large data sets from multiple sources
Source: BMC Med Inform Decis Mak. 2012 Jun 28;12:59. doi: 10.1186/1472-6947-12-59 (PMC3439324; doi:10.1186/1472-6947-12-59)
Supplement: Additional file 1 — Detailed generation of the simulated data[53]. [file 1472-6947-12-59-S1.pdf]

APPENDIX A: SIMULATED DATA GENERATION

A synthetic data generator is developed following realistic characteristics, such as distribution and errors, of which the errors, or modifications, are typographic errors (insertion, deletion, substitution, or transportation of adjacent characters), optical character recognition errors based on shape similarity, and phonetic errors based on sound similarity. [54].

We used that data generator to generate three simulated data sets of size *1,000*, *5,000*, and *10,000*. The details of each data set are as follows. 1) data set of size *1,000*: it contains *500* original records and *500* duplicated records, of which each original record has one duplicated records with at most four modifications in one field and at most six modifications in one record; 2) data set of size *5,000*: it contains *2,000* original records and *3,000* duplicated records, of which the modifications occurs the same as size *1,000* and each original record can have at most five duplicates following Zipf Distribution; 3) data set of size *10,000*: it contains *7,500* original records and *2,500* duplicated records, of which the modifications occurs the same as size *1,000* and each original record can have at most five duplicates following Poisson Distribution. Therefore, in the three data sets, it becomes more and more challenging not only in the size but only in the complexity of modifications; 4) a forth data set with *1,000,000* records is a collection of *100* copies of the third one.
