# Supplementary material for: Efficient algorithms for fast integration on large data sets from multiple sources
Source: BMC Med Inform Decis Mak. 2012 Jun 28;12:59. doi: 10.1186/1472-6947-12-59 (PMC3439324; doi:10.1186/1472-6947-12-59)
Supplement: Additional file 2 — Introduction to the real data. [file 1472-6947-12-59-S2.pdf]

APPENDIX B: INTRODUCTION TO REAL DATA

We have used four real datasets from Connecticut State agencies (Quick Recovery and Quick Recovery Medical, eCAMRIS, and Level of Need), two from the University of Connecticut's Dental Clinic (UCHC) and two from the Connecticut Department of Developmental Services (DDS). Data sets are described below.

Quick Recovery (QR) is the clinical database at the University of Connecticut Health Center, which has been in use since 2003 for scheduling, treatment planning, billing and reporting for the over 200k patients seen at the University of Connecticut School of Dental Medicine and Dental Clinics. QR is with 1,002,342 records for dentally billed procedures, and an additional 2,116 records for medically billed procedures (Quick Recovery Medical). The database contains patient demographics as well as information on procedures, financials and providers. For this project only demographic data were used. Available demographic data include first and last name and address as well as gender, Social Security number, date of birth, University of Connecticut Health Center Medical Record Number, and Social Security Number.

eCAMRIS is the State of Connecticut Department of Developmental Service's client database which has been used since 1989 to track clients and their associations with placements and providers. eCAMRIS contains information on client demographics, diagnoses and placements as well as information on DDS providers. For this project we used the demographic table which is an individual level table that contains 31,984 records. Identifiers consistently available in this table are the first name, last name, gender and an internally assigned DDS identification number. Also available inconsistently are middle name, address and Social Security Number.

Level of Need (LON) has been used by DDS since 2006 to assess client level of need and assists with client planning and resource allocation. LON contains over 400 question assessments concerning overall need and health status of 12,000 DDS clients. Assessments are updated for most DDS clients yearly but are updated more often if client status changes. LON has three tables, one of assessment information, a second of question answers, and a third of results of algorithms aimed at addressing specific areas of need. For this project we used the assessments table which is an assessment level table containing 47, 436 records. Consistently available identifiers are the first and last names, date of birth and an internally assigned DDS identification number. Also available for some assessments is middle name.
